# Complete Sets of Radiating and Nonradiating Parts of a Source and Their Fields with Applications in Inverse Scattering Limited-Angle Problems

**DOI:** 10.1155/IJBI/2006/93074

**Published:** 2007-01-09

**Authors:** E. Wallacher, A. K. Louis

**Affiliations:** Fakultät für Mathematik und Informatik, Universität des Saarlandes, Saarbrücken 66041, Germany

## Abstract

Many algorithms applied in inverse scattering problems use source-field systems instead of the direct computation of the unknown scatterer. It is well known that the resulting source problem does not have a unique solution, since certain parts of the source totally vanish outside of the reconstruction area. This paper provides for the two-dimensional case special sets of functions, which include all radiating and all nonradiating parts of the source. These sets are used to solve an acoustic inverse problem in two steps. The problem under discussion consists of determining an inhomogeneous obstacle supported in a part of a disc, from data, known for a subset of a two-dimensional circle. In a first step, the radiating parts are computed by solving a linear problem. The second step is nonlinear and consists of determining the nonradiating parts.


Hindawi Publishing Corporation
International Journal of Biomedical Imaging
Volume 2006, Article ID 93074, Pages 1–13
DOI 10.1155/IJBI/2006/93074

### Complete Sets of Radiating and Nonradiating Parts of a Source and Their Fields with Applications in Inverse Scattering Limited-Angle Problems

**E. Wallacher and A. K. Louis**

*Fakultät für Mathematik und Informatik, Universität des Saarlandes, 66041 Saarbrücken, Germany*

Received 24 March 2006; Revised 12 October 2006; Accepted 12 October 2006

Recommended for Publication by Freek Beekman

Many algorithms applied in inverse scattering problems use source-field systems instead of the direct computation of the unknown scatterer. It is well known that the resulting source problem does not have a unique solution, since certain parts of the source totally vanish outside of the reconstruction area. This paper provides for the two-dimensional case special sets of functions, which include all radiating and all nonradiating parts of the source. These sets are used to solve an acoustic inverse problem in two steps. The problem under discussion consists of determining an inhomogeneous obstacle supported in a part of a disc, from data, known for a subset of a two-dimensional circle. In a first step, the radiating parts are computed by solving a linear problem. The second step is nonlinear and consists of determining the nonradiating parts.

Copyright © 2006 E. Wallacher and A. K. Louis. This is an open access article distributed under the Creative Commons Attribution License, which permits unrestricted use, distribution, and reproduction in any medium, provided the original work is properly cited.

### 1. INTRODUCTION

Medical imaging involves the solution of mathematical inverse problems. This means that cause (the properties of living tissue) is inferred from effect (the observed signal). In the case of ultrasound tomography, the probe consists of ultrasonic pressure waves, and echoes inside the tissue show the internal structure.

The properties of the tissue inside a body are described by the function

$$f = \frac{c_0^2}{c^2} - 1 + \frac{i2ac_0}{kc}, \tag{1}$$

where $c$ is the local speed of sound. Outside the body the speed $c = c_0$ is constant. The function $a$ comprises the attenuation of a wave. The wavenumber $k$ depends on the frequency $\omega$ of the ultrasound scanner. They are simply related by the equation

$$k = \frac{\omega}{c_0}. \tag{2}$$

Outside the body the function $f$ becomes zero. Therefore, let us assume that the support of $f$ is a subset of $\Omega = \{x \in \mathbb{R}^2 : |x| < R\}$.

Furthermore, the incident wave $u_i$, which is generated by the ultrasound scanner, solves the homogeneous wave equation

$$(\triangle + k^2)u_i = 0. \tag{3}$$

The scattered field $u_s$, which is generated by the scatterer $f$ and the incident wave $u_i$, is described by the Lippmann-Schwinger integral equation

$$u_s(x) = k^2 \int_\Omega G(x,y) f(y)\big(u_i(y) + u_s(y)\big)\,dy, \tag{4}$$

where $G$ is the Green function associated with the Helmholtz operator $(\triangle + k^2)$ and the Sommerfeld radiation condition in $\mathbb{R}^2$,

$$\lim_{r\to\infty} r^{1/2}\left(\frac{\partial u_s}{\partial r} - iku_s\right) = 0, \quad r = \|x\|. \tag{5}$$

The total field $u$,

$$u = u_i + u_s, \tag{6}$$

is determined by the sum of the incident wave and the scattered field. Equation (4) is valid for all $x \in \mathbb{R}^2$. In the

2-dimensional case,

$$G(x, y) = \frac{i}{4} H_o(k\|x - y\|), \tag{7}$$

where $H_o$ is the Bessel function of the third kind of order 0 (see, e.g., [1]). In order to formulate the inverse problem, we assume that the data $g := u_s|_\Gamma$ is known for a subset of the boundary $\Gamma \subset \partial\Omega$ of the reconstruction area. If the operator $A$ is defined as

$$\begin{gathered} A : L_2(\Omega) \longrightarrow L_2(\Gamma), \\ A\Psi(x) = k^2 \int_\Omega G(x, y)\Psi(y)dy, \end{gathered} \tag{8}$$

then the inverse problem consists in solving the integral equation

$$A(f(u_i + u_s)) = g, \tag{9}$$

which is a nonlinear problem, since $f$ and $u_s$ are unknown in the interior of the domain $\Omega$. To get rid of the nonlinearity, this problem is reformulated as a source problem by defining

$$\Phi := f(u_i + u_s). \tag{10}$$

Now the problem reads as

$$A\Phi = g. \tag{11}$$

Denote by $B$ the operator

$$\begin{gathered} B : L_2(\Omega) \longrightarrow L_2(\Omega), \\ B\Psi(x) = k^2 \int_\Omega G(x, y)\Psi(y)dy, \end{gathered} \tag{12}$$

then

$$u_s = B\Phi \tag{13}$$

is the field generated by the source $\Phi$.

It is well known that the solution of the source problem (11) is not unique due to the existence of some nonradiating parts of the function $\Phi$ which completely vanish outside of the region $\Omega$. In Sections 2 and 4, complete sets of functions of the radiating and the nonradiating parts of the source $\Phi$ are constructed. This decomposition already has successfully been used in [2] to solve the inverse scattering problem, where the authors discretise the operator $A$ and computed the singular value decomposition numerically, that contains the radiating and the nonradiating parts, respectively.

To get uniqueness of the reconstruction problem, multiple experiments have to be performed using pairwise different incident waves $u_i^m$ (see [3]). Then the corresponding sources are related by

$$\frac{\Phi^m}{B\Phi^m + u_i^m} = f = \frac{\Phi^n}{B\Phi^n + u_i^n}, \tag{14}$$

which follows from (10). It has been shown, for example, in [4, 5], that there exists a unique solution $f$ using incident plane waves $u_i^\alpha(x) = e^{ik\langle\alpha,x\rangle}$ if data are collected for every direction $\alpha$ of the unit sphere $S^1$. This proves the existence of a unique solution for every $\Phi^\alpha$ for all $\alpha \in S^1$ in this case.

In Section 2, complete sets of radiating and nonradiating sources for the case $\Gamma = \partial\Omega$ are constructed. The corresponding fields to these sets are computed in Section 3. In Section 4, the limited angle problem $\Gamma \subset \partial\Omega$ is considered. Complete sets of radiating and nonradiating sources are developed by using the singular value decomposition (SVD) of the operator $A$. Algorithms and strategies, which use the decomposition of the sources to solve the inverse problem, are given in Section 5. In the last section, numerical results are presented.

### 2. COMPLETE SETS OF RADIATING AND NONRADIATING SOURCES

In this section, it is assumed that the data are given on the complete boundary, that is, $\Gamma = \partial\Omega$. The general case $\Gamma \subset \partial\Omega$ will be discussed in Section 4. Some efforts were made in [6] to construct complete sets of nonradiating sources. The authors used the differential equation

$$(\triangle + k^2)u_s = -k^2 f(u_s + u_i), \tag{15}$$

which is equivalent to the integral equation (4). They determined a basis of functions for radial symmetric nonradiating sources. As an alternative approach in the current paper the integral equation is considered to get a complete set of nonradiating sources.

From (15) and the definition of the source (10), it follows that a field $u_s$ generated by a source $\Phi \in L_2(\Omega)$, which is zero at the boundary, completely vanishes outside of $\Omega$. Therefore, $A\Phi = 0$ is a necessary and sufficient condition for $\Phi$ to be a nonradiating source. Now the problem is to determine a complete set of the null space $N(A)$, which contains all nonradiating sources. On the other hand, a basis of $N(A)^\perp$ represents a complete set of the radiating parts of the source.

A useful tool for investigating an operator is the singular value decomposition (SVD) $(v_m, u_m, \sigma_m)$, which was determined in [7] in the case $\Gamma = \partial\Omega$. The singular functions $v_m$ and $u_m$ are given up to constants by

$$\begin{gathered} v_m(r\omega) = J_m(kr)Y_m(\omega), \\ u_m(R\omega) = Y_m(\omega). \end{gathered} \tag{16}$$

The functions $J_m$ denote the Bessel functions of the first kind of order $m$, and $Y_m$ are the spherical harmonics of degree $|m|$, which are in the 2-dimensional case given by $Y_m(\omega(\varphi)) = e^{im\varphi}$, where $\omega(\varphi)$ are vectors of the unit sphere $S^1$ depending on the angle $\varphi$. The singular functions $v_m$ generate an orthogonal basis of the space $N(A)^\perp$ and hence $\{v_m\}_{m\in\mathbb{Z}}$ is a complete set of radiating sources. To complete this set to a base of $L_2(\Omega)$ the null space $N(A)$, which includes all nonradiating functions, has to be characterized. Assuming that a nonradiating source can be separated in a part $q$, which only depends on the radius, and a part $Y_p$, depending only on the direction of a point $x = r\omega \in \Omega$, $\Phi(r\omega) = q(r)Y_p(\omega)$, then the function $\Phi$ has to be orthogonal to all functions $v_m \in N(A)^{\perp}$. That means

$$\langle \Phi, v_m \rangle = 0 \quad \forall m \in \mathbb{Z}. \tag{17}$$

Introducing polar coordinates one can deduce

$$\int_0^R rq(r)J_m(kr)\int_{S^1} Y_p(\omega)\overline{Y_m(\omega)}d\omega\, dr = 0. \tag{18}$$

Finally, the orthogonality property

$$\int_{S^1} Y_m(\omega)\overline{Y_p(\omega)}\, d\omega = 2\pi\delta_{m,p} \tag{19}$$

of the spherical harmonics leads to the conclusion that

$$\int_0^R rq(r)J_p(kr)dr = 0 \tag{20}$$

has to be fulfilled for $\Phi$ to be a nonradiating source.

The problem changes into finding sets of functions $\{q_{p,n}\}_{n\in\mathbb{N}}$, that provide in combination with the function $J_p(k\cdot)$ a basis of the Hilbert space $L_2([0,R], w^{-1})$ with the weight $w(r) = r$. A helpful tool to construct such systems is given by a special case of Lommel's theorem (see, e.g., [8]), which is summarized in Appendix 7. Now it is possible to get an orthogonal basis of $L_2(\Omega)$, which includes all radiating parts $\{v_m\}_{m\in\mathbb{Z}}$. The existence of the zeros, claimed in the theorem below, follows from Appendix 7 1–4. From formula 5 in Appendix 7 one can deduce that the number of zeros is countable.

**Theorem 1.** *Let $k, R \in \mathbb{R}^+$. For every $m \in \mathbb{Z}$ denote*

$$a_m = \begin{cases} \dfrac{-kRJ_m'(kR)}{J_m(kR)} & \text{if } J_m(kR) \neq 0, \\ 1 & \text{otherwise}, \end{cases} \qquad b_m = \begin{cases} 1 & \text{if } J_m(kR) \neq 0, \\ 0 & \text{otherwise}. \end{cases} \tag{21}$$

*Furthermore, let $\lambda_{m,n}$ ($n \in \mathbb{N}$) be the positive zeros of $a_mJ_m(r) + b_mrJ_m'(r)$ (in the case where $a + |m| < 0$, any of the two zeros in $\{ix \mid x \in \mathbb{R}^+\}$ are to be added). After permutation let $\lambda_{m,o} = kR$. Then the functions*

$$w_{m,n}(r\omega) := J_m\left(\frac{\lambda_{m,n}}{R}r\right)Y_m(\omega) \tag{22}$$

*build an orthogonal basis of $L_2(\Omega)$ and especially*

$$w_{m,o}(r\omega) = J_m(kr)Y_m(\omega). \tag{23}$$

*Proof.* It follows from Lommel's theorem (see Appendix 7) and the orthogonality property (19) of the spherical harmonics that the functions $w_{m,n}$ are orthogonal on $L_2(\Omega)$. The fact that $\lambda_{m,o} = kR$ is a zero of $a_mJ_m(r) + b_mrJ_m'(r)$ follows from the definition of $a_m$ and $b_m$. Therefore, the functions $w_{m,o}(r\omega)$ are equal to $J_m(kr)Y_m(\omega)$ and thus the radiating parts are all contained in the basis.

To complete the proof, it has to be shown that for any $m \in \mathbb{Z}$ the set $\{q_{m,n}(r)\}_{n\in\mathbb{N}} := \{J_m((\lambda_{m,n}/R)r)\}_{n\in\mathbb{N}}$ is a complete basis for $L_2([0,R], w^{-1})$. For the case $b_m = 0$ this was shown in [9]. In a similar way it can also be proved for the case $b_m = 1$. A detailed proof is given in [10].

Now let $g \in L_2(\Omega)$ be any function, that is orthogonal to every function $w_{m,n}$. A consequence of the completeness of the spherical harmonics $\{Y_m\}_{m\in\mathbb{Z}}$ on the space $L_2(S^1)$ and the completeness of the functions $\{q_{m,n}\}_{n\in\mathbb{N}}$ in $L_2([0,R], w^{-1})$ is that $g$ has to be zero, which finishes the proof. □

Sometimes it is better to deal with normalized functions. An approximation can always be computed by numerical integration. But in this case one can also do this analytically, which follows from Theorem 2 below.

**Theorem 2.** *Let $\{w_{m,n}\}_{m\in\mathbb{Z},\, n\in\mathbb{N}}$ be the orthogonal basis defined in Theorem 1. Then, the norm of $w_{m,n}$ is given in the case $b_m = 1$ by*

$$||w_{m,n}|| = \frac{\sqrt{\pi}R}{|\lambda_{m,n}|}\sqrt{[a_m^2 + \lambda_{m,n}^2 - m^2]}\,|J_m(\lambda_{m,n})|, \tag{24}$$

*and if $b_m = 0$,*

$$||w_{m,n}|| = \frac{\sqrt{\pi}R}{|\lambda_{m,n}|}\,|J_m'(\lambda_{m,n})|. \tag{25}$$

*Proof.* Applying Lommel's theorem (see Appendix 7) and the orthogonality properties (19) of the spherical harmonics leads directly to the norms of $w_{m,n}$. □

In the next theorem the main results of this section are resumed.

**Theorem 3.** *Let $\{w_{m,n}\}_{m\in\mathbb{Z},\, n\in\mathbb{N}}$ be the orthogonal base of $L_2(\Omega)$ defined in Theorem 1, then*

(i) $\{w_{m,o} \mid m \in \mathbb{Z}\}$ *is a complete orthogonal set of radiating sources,*
(ii) $\{w_{m,n} \mid m \in \mathbb{Z},\ 1 \leq n \in \mathbb{N}\}$ *is a complete orthogonal set of nonradiating sources.*

*Proof.* Let $w_{m,n}$ denote the functions of Theorem 1. Because of the singular value decomposition (16) of the operator ,it is obvious that (i) holds. Again it is a consequence of Theorem 1 that (ii) is also true. □

### 3. THE CORRESPONDING FIELDS AND PROPERTIES

Let $w_{m,n}$ be the functions of the orthogonal base of Theorem 1, then they are associated to the radiating and nonradiating parts of a source as described in Theorem 3. To develop an algorithm that solves the inverse problem, the corresponding fields $u_{m,n}$ of the sources $w_{m,n}$ have to be computed. They are related by (13) $u_{m,n} = Bw_{m,n}$.

**Theorem 4.** *The fields $u_{m,n}$ corresponding to the sources $w_{m,n}$ are given by*

$$u_{m,n}(r\omega) = \frac{ik^2\pi}{2}\left[H_m(kr)I^1_{m,n}(r) + J_m(kr)I^2_{m,n}(r)\right]Y_m(\omega), \tag{26}$$

*where*

$$I^1_{m,n}(r) = \begin{cases} \dfrac{r}{k^2-(\lambda_{m,n}/R)^2}\left[kJ_{m+1}(kr)J_m\left(\dfrac{\lambda_{m,n}}{R}r\right) - \dfrac{\lambda_{m,n}}{R}J_m(kr)J_{m+1}\left(\dfrac{\lambda_{m,n}}{R}r\right)\right], & n \geq 1, \\ \dfrac{1}{2k^2}\left[r^2k^2(J'_m(kr))^2 + ([kr]^2-m^2)J^2_m(kr)\right], & n = 0, \end{cases}$$

$$I^2_{m,n}(r) = \begin{cases} \dfrac{s}{k^2-(\lambda_{m,n}/R)^2}\left[kH_{m+1}(ks)J_m\left(\dfrac{\lambda_{m,n}}{R}s\right) - \dfrac{\lambda_{m,n}}{R}H_m(ks)J_{m+1}\left(\dfrac{\lambda_{m,n}}{R}s\right)\right]\Bigg|_r^R, & n \geq 1, \\ \dfrac{1}{2k^2}\left[s^2k^2H'_m(ks)J'_m(ks) + ([ks]^2-m^2)H_m(ks)J_m(ks)\right]\Big|_r^R, & n = 0. \end{cases} \tag{27}$$

*Proof.* Using the expansion of Hankel function (see Appendix 7 formula 10) and Lommel's theorem (see Appendix 7) leads to the statement given in Theorem 4. □

A more detailed examination shows that the fields $u_{m,n}$ corresponding to the nonradiating sources $w_{m,n}$ ($n \geq 1$) can be expressed by the functions $w_{m,n}$ themselves in the following way.

**Theorem 5.** *The fields $u_{m,n}$ caused by the nonradiating sources $w_{m,n}$ ($n \geq 1$) have the form*

$$u_{m,n}(r\omega) = d_{m,n}w_{m,o}(r\omega) + e_{m,n}w_{m,n}(r\omega), \tag{28}$$

*where*

$$d_{m,n} = \frac{ik^2\pi}{2(k^2-(\lambda_{m,n}/R)^2)}\left[kRH_{m+1}(kR)J_m(\lambda_{m,n}) - \lambda_{m,n}H_m(kR)J_{m+1}(\lambda_{m,n})\right],$$

$$e_{m,n} = \frac{-k^2}{k^2-(\lambda_{m,n}/R)^2} \tag{29}$$

*are constants.*

*Proof.* Let $I^1_{m,n}(r)$ and $I^2_{m,n}(r)$ be the functions due to Theorem 4. From Theorem 1, we obtain

$$Bw_{m,n} = \sum_{p\in\mathbb{Z}}\sum_{q\in\mathbb{N}}\left\langle Bw_{m,n}, \frac{w_{p,q}}{||w_{p,q}||^2}\right\rangle w_{p,q},$$

$$\begin{aligned} \frac{2}{ik^2\pi}\left\langle Bw_{m,n}, \frac{w_{p,q}}{||w_{p,q}||^2}\right\rangle &= \frac{1}{||w_{p,q}||^2}\int_o^R r\left[H_m(kr)I^1_{m,n}(r) + J_m(kr)I^2_{m,n}(r)\right]J_p \\ &\quad\times\left(\frac{\lambda_{p,q}}{R}r\right)\underbrace{\int_{S^1}Y_m(\omega)\overline{Y_p(\omega)}\,d\omega}_{=2\pi\delta_{m,p}}\,dr \\ &= \frac{2\pi\delta_{m,p}}{(k^2-(\lambda_{m,n}/R^2)^2)||w_{m,q}||^2} \\ &\quad\times\left[\int_o^R rT_m(r)J_m\left(\frac{\lambda_{m,n}}{R}r\right)J_m\left(\frac{\lambda_{m,q}}{R}r\right) + rS_m(R)J_m(kr)J_m\left(\frac{\lambda_{m,q}}{R}r\right)dr\right], \end{aligned} \tag{30}$$

where

$$\begin{aligned} T_m(r) &= kr\left[H_m(kr)J_{m+1}(kr) - J_m(kr)H_{m+1}(kr)\right], \\ S_m(R) &= kRH_{m+1}(kR)J_m(\lambda_{m,n}) - \lambda_{m,n}H_m(kR)J_{m+1}(\lambda_{m,n}). \end{aligned} \tag{31}$$

Applying formulas 9 and 11 of Appendix 7 leads to

$$T_m(r) = \frac{2i}{\pi}. \tag{32}$$

Inserting $T_m$ and $S_m$ yields

$$\begin{aligned} \frac{k^2-(\lambda_{m,n}/R)^2}{ik^2\pi^2c_{m,n}c_{p,q}}\left\langle Bw_{m,n}, \frac{w_{p,q}}{||w_{p,q}||^2}\right\rangle &= \frac{2i\delta_{m,p}}{\pi||w_{m,q}||^2}\underbrace{\int_o^R rJ_m\left(\frac{\lambda_{m,n}}{R}r\right)J_m\left(\frac{\lambda_{m,q}}{R}r\right)dr}_{(\|w_{m,n}\|^2/2\pi)\delta_{n,q}} \\ &\quad+ \frac{S_m(R)\delta_{m,p}}{||w_{m,q}||^2}\underbrace{\int_o^R rJ_m(kr)J_m\left(\frac{\lambda_{m,q}}{R}r\right)dr}_{(\|w_{m,o}\|^2/2\pi)\delta_{o,q}}. \end{aligned} \tag{33}$$

From this we deduce that

$$\langle Bw_{m,n}, w_{p,q}\rangle = \frac{-k^2}{k^2-(\lambda_{m,n}/R)^2}\delta_{m,p}\delta_{n,q} + \frac{ik^2\pi}{2\left(k^2-(\lambda_{m,n}/R)^2\right)}S_m(R)\delta_{m,p}\delta_{o,q}. \quad (34)$$

□

Finally, the properties of the source-field systems $(u_{m,n}, w_{m,n})$ are summarized in the next theorem.

**Theorem 6.** *Let $u_{m,n}$ and $w_{m,n}$ be the fields and sources defined as above. Then,*

(i) $Bw_{m,n} = u_{m,n}$,
(ii) $(\triangle + k^2)u_{m,n} = -k^2 w_{m,n}$,
(iii) $(\triangle + k^2)w_{m,n} = (k^2 - \lambda_{m,n}^2/R^2)w_{m,n}$,
(iv) $[(\triangle + k^2)]^2 u_{m,o} = 0$,
(v) *the space of the radiating sources is orthogonal to the space of nonradiating sources.*

These properties were proved in [11] for any complex-valued or vector source-field system described by a linear scalar or vector partial differential equation. In this case, they follow immediately from the outlines made before and the fact that the functions $w_{m,n}$ are eigenfunctions of the Laplace operator with eigenvalues $-(\lambda_{m,n}/R)^2$.

### 4. THE LIMITED-ANGLE PROBLEM

The singular value decomposition (SVD) is a powerful tool to analyze a problem. The range and the orthogonal complement of the nullspace of an operator is given by the singular functions. The behavior of the singular values characterizes the ill-posedness of the problem. A classification is, for example, outlined in [12]. The goal of this section is to derive the SVD of the operator $A$ for the general case $\Gamma \subset \partial\Omega$. To handle this problem, it is assumed that $\Gamma$ is parameterized in the following way:

$$\Gamma_{\eta(\alpha),\beta} = \{ \mathrm{R}e^{i\delta} \mid \delta \in [\eta(\alpha)-\beta;\eta(\alpha)+\beta]\}, \quad (35)$$

where $\eta$ is an arbitrary function defined on the sphere

$$\eta : [0;2\pi) \longrightarrow [0;2\pi). \quad (36)$$

The measurement setup is shown in Figure 1. In this case, the center of the detector is $\eta(\alpha)$ and the length of the detector is $2\beta$. The operator under consideration depends on the direction of the incident wave as well as of the length of the detector. The function $\eta$ only ensures that the center of the detector is independent of the direction of the incident wave $u_i^\alpha$. For instance, one can imagine that for applications the detector can always be installed in the opposite of the incident wave. That means $\eta(\alpha) = \alpha$. On the other hand, it is possible that the detector is fixed somewhere and so $\eta(\alpha) \equiv c$ becomes a constant. For the sake of simplicity, we replace in the following $\eta(\alpha)$ by $\alpha$ because the arguments in the prove

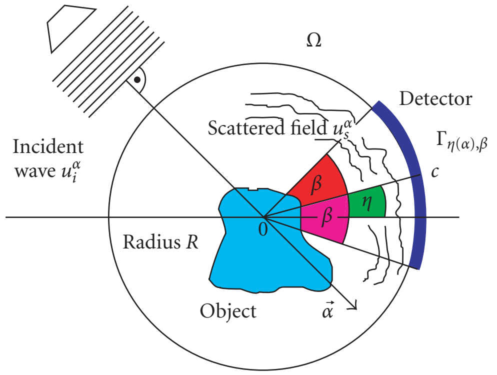

Figure 1: Measurement setup.

are the same:

$$A^{\alpha,\beta} : L_2(\Omega) \longrightarrow L_2(\Gamma_{\alpha,\beta}), \qquad A^{\alpha,\beta}\Psi(x) = k^2 \int_\Omega G(x,y)\Psi(y)dy. \quad (37)$$

To get the SVD of the operator $A := A^{\alpha,\beta}$, the eigenfunctions and eigenvalues of the operator $A^*A$ have to be computed. Therefore, a suitable representation of this operator is needed. In the following, $\alpha$ is considered as the same time as a vector on the sphere $S^1$ as well as the corresponding angle.

**Theorem 7.** *Let $A$ be the operator defined in (37) for an arbitrary $\alpha \in [0,2\pi)$ and $\beta \in (0,\pi]$. (Let $f \in L_2(\Omega)$ and $w_{m,n}$ ($m \in \mathbb{Z}$, $n \in \mathbb{N}$) be the $L_2$-basis of Theorem 1.) Then,*

$$(A^*Af)(r\omega) = \sum_{m\in\mathbb{Z}}\sum_{n\in\mathbb{Z}} e^{i(n-m)\alpha}S(m,n,\beta)\left\langle f, \frac{w_{n,o}}{||w_{n,o}||}\right\rangle \frac{w_{m,o}(r\omega)}{||w_{m,o}||}, \quad (38)$$

*where*

$$S(m,n,\beta) = \frac{\beta|k^2|^2 R}{8}\overline{H_m(kR)}H_n(kR)||w_{m,o}||\,||w_{n,o}||\,\mathrm{sinc}\,(\beta(n-m)) \quad (39)$$

*is a weighted Sinc matrix.*

*Proof.* We have the identity

$$\langle Af,g\rangle = \int_{\Gamma_{\beta,\alpha}}\int_\Omega k^2G(k\|x-y\|)f(y)dy\overline{g(x)}\,dx = \int_\Omega\int_{\Gamma_{\beta,\alpha}} \overline{\overline{k^2G(k\|x-y\|)}g(x)dx}f(y)dy = \langle f,A^*g\rangle. \quad (40)$$

Thus, we obtain

$$(A^*Af)(x) = \overline{k^2}\int_{\Gamma_{\beta,\alpha}} \overline{G(k\|x-y\|)}k^2 \times \int_{\Omega} G(k\|y-z\|)f(z)dz\,dy \overset{(7)}{=} \frac{|k^2|^2}{16}\int_{\Gamma_{\beta,\alpha}} \overline{H_0(k\|x-y\|)} \times \int_{\Omega} H_0(k\|y-z\|)f(z)dz\,dy. \quad (41)$$

Using polar coordinates $x = r_x\omega_x$ and the series representation of the Hankel function (see Appendix 7 formula 10), one can deduce

$$(A^*Af)(r_x\omega_x) = \frac{|k^2|^2 R}{16}\int_{\omega(\Gamma)} \sum_{m\in\mathbb{Z}} \overline{H_m(kR)Y_m(\omega_y)J_m(kr_x)}Y_m(\omega_x) \times \int_0^R \int_{S^1} r_z \sum_{n\in\mathbb{Z}} H_n(kR)Y_n(\omega_y) \times J_n(kr_z)\overline{Y_n(\omega_z)}f(r_z\omega_z)d\omega_z\,dr_z\,d\omega_y. \quad (42)$$

Uniform convergence leads finally to

$$(A^*Af)(r_x\omega_x) = \frac{|k^2|^2 R}{16}\sum_{m\in\mathbb{Z}}\sum_{n\in\mathbb{Z}} \overline{H_m(kR)}\underbrace{J_m(kr_x)Y_m(\omega_x)}_{w_{m,o}(r_x\omega_x)}H_n(kR) \times \underbrace{\int_{\omega(\Gamma)} \overline{Y_m(\omega_y)}Y_n(\omega_y)d\omega_y}_{e^{i(n-m)\alpha}2\beta\,\mathrm{sinc}(\beta(n-m))} \times \underbrace{\int_0^R r_z J_n(kr_z)\int_{S^1}\overline{Y_n(\omega_z)}f(r_z\omega_z)dr_z d\omega_z}_{\langle f, w_{n,o}\rangle} = \sum_{m\in\mathbb{Z}}\sum_{n\in\mathbb{Z}} e^{i(n-m)\alpha}S(m,n,\beta)\left\langle f, \frac{w_{n,o}}{\|w_{n,o}\|}\right\rangle \frac{w_{m,o}(r_x\omega_x)}{w_{m,o}}. \quad (43)$$

□

Some useful properties of the Sinc-matrix $(S(m,n,\beta))_{m,n\in\mathbb{Z}}$ are summarized in the following lemma.

**Lemma 1.** *By using the notation above, the following statements hold:*

(i) $a_m = a_{-m}$;
(ii) $\lambda_{m,n} = \lambda_{-m,n}$;
(iii) $\|w_{m,n}\| = \|w_{-m,n}\|$;
(iv) $S(m,n,\beta) = S(-m,-n,\beta)$;
(v) $S(m,n,\beta) = \overline{S(n,m,\beta)}$;
(vi) *the operator* $(S(m,n,\beta))_{m,n\in\mathbb{Z}} : l^2(\mathbb{Z}) \to l^2(\mathbb{Z})$ *is selfadjoint and compact.*

*Proof.* Assumption (i) and (ii) follow immediately from

$$J_m(r) = (-1)^m J_{-m}(r). \quad (44)$$

*Assumption (iii)*

$$\|w_{m,n}\| = \left(\frac{\pi R^2}{\lambda_{m,n}^2}[a_m^2 + \lambda_{m,n}^2 - m^2]J_m^2(\lambda_{m,n})\right)^{1/2} \overset{(i)+(ii)}{=} \left(\frac{\pi R^2}{\lambda_{-m,n}^2}[a_{-m}^2 + \lambda_{-m,n}^2 - (-m)^2]J_{-m}^2(\lambda_{-m,n})\right)^{1/2} = \|w_{-m,n}\|. \quad (45)$$

*Assumption (iv)*

Because of $H_m(kR) = (-1)^m H_{-m}(kR)$ and $\mathrm{sinc}(t) = \mathrm{sinc}(-t)$, one has

$$S(m,n,\beta) = \frac{\beta|k^2|^2 R}{8}\overline{H_{-m}(kR)}H_{-n}(kR)\|w_{-m,o}\| \times \|w_{-n,o}\|\,\mathrm{sinc}(\beta(-n+m)) = S(-m,-n,\beta). \quad (46)$$

*Assumption (v)*

Only the Hankel functions possess a complex-valued part in $S(m,n,\beta)$. Therefore,

$$S(m,n,\beta) = \overline{\frac{\beta|k^2|^2 R}{8}H_m(kR)\overline{H_n(kR)}\|w_{m,o}\|\,\|w_{n,o}\|\,\mathrm{sinc}(\beta(n-m))} = \overline{S(n,m,\beta)}. \quad (47)$$

*Assumption (vi)*

Let $C = (S(m,n,\beta))_{m,n\in\mathbb{Z}}$ and $W = \overline{\mathrm{span}\{w_{m,o} \mid m\in\mathbb{Z}\}} \subset L_2(\Omega)$. Then the functions $(w_{m,o})_{m\in\mathbb{Z}}$ are an orthogonal basis of the Hilbert-space W and therefore the function

$$\iota : l^2(\mathbb{Z}) \longrightarrow W(x_m)_{m\in\mathbb{Z}} \longmapsto \sum_{m\in\mathbb{Z}} x_m \frac{w_{m,o}}{\|w_{m,o}\|} \quad (48)$$

is an isomorphism. One can conclude that

$$C(x_m)_{m\in\mathbb{Z}} = \iota^{-1}\iota C(x_m)_{m\in\mathbb{Z}},$$
$$C(x_m)_{m\in\mathbb{Z}} = \iota^{-1}\iota\left(\sum_{n\in\mathbb{Z}} S(m,n,\beta)x_n\right)_{m\in\mathbb{Z}},$$
$$C(x_m)_{m\in\mathbb{Z}} = \iota^{-1}\left(\sum_{m\in\mathbb{Z}}\sum_{n\in\mathbb{Z}} S(m,n,\beta)x_n\frac{w_{m,o}}{\|w_{m,o}\|}\right), \quad (49)$$
$$C(x_m)_{m\in\mathbb{Z}} \overset{\alpha=0}{=} \iota^{-1}\left(A^*A\left(\sum_{m\in\mathbb{Z}} x_m\frac{w_{m,o}}{\|w_{m,o}\|}\right)\right),$$
$$C(x_m)_{m\in\mathbb{Z}} = \iota^{-1}\underbrace{(A^*A\iota(x_m)_{m\in\mathbb{Z}})}_{\in W} \in l^2(\mathbb{Z}).$$

Hence $C$ is well defined. From (v) it follows immediately that $C$ is selfadjoint. It is well known that $A$ is a compact operator and thus $A^*A$ is compact. From (49) one can deduce that C is also compact. □

Now it is possible to compute the SVD of the operator $A$.

**Theorem 8.** *Let $(v^{\beta}_{p,m})_{m\in\mathbb{Z}}$ $(p \in \mathbb{Z})$ be the orthonormalized eigenvectors of the matrix $(S(m,n,\beta))_{m,n\in\mathbb{Z}}$ to the eigenvalues $\lambda^{\beta}_p$ (the existence follows from Lemma 1(vi)). The normalization of $v^{\beta}_{p,m}$ holds for the inner product on $l^2(\mathbb{Z})$, that is, $\|(v^{\beta}_{p,m})_{m\in\mathbb{Z}}\|^2_{l^2} = \sum_{m\in\mathbb{Z}} |v^{\beta}_{p,m}|^2$. Then the operator $A^{\alpha,\beta}$ has the SVD $(v^{\alpha,\beta}_p, u^{\alpha,\beta}_p, \sigma^{\beta}_p)_{p\in\mathbb{Z}}$ with*

$$\sigma^{\beta}_p = \sqrt{\lambda^{\beta}_p},$$
$$v^{\alpha,\beta}_p(r\omega) = \sum_{m\in\mathbb{Z}} v^{\beta}_{p,m} e^{-im\alpha} \frac{w_{m,o}(r\omega)}{\|w_{m,o}\|}, \tag{50}$$
$$u^{\alpha,\beta}_p(R\omega) = \frac{ik^2}{4\sigma^{\beta}_p} \sum_{m\in\mathbb{Z}} v^{\beta}_{p,m} H_m(kR)\|w_{m,o}\| e^{-im\alpha} Y_m(\omega).$$

*Proof.* For the sake of simplicity, the dependence of the parameters $\alpha$ and $\beta$ is dropped for the SVD in this proof. It is shown in Lemma 1 that the operator $(S(m,n,\beta))_{m,n\in\mathbb{Z}}$ is selfadjoint and compact. This fact provides the existence of an orthonormal basis of eigenfunctions,

$$\begin{aligned}
(A^*Av_p)(r\omega) &= \sum_{m\in\mathbb{Z}} \sum_{n\in\mathbb{Z}} e^{i(n-m)\alpha} S(m,n,\beta) \\
&\quad \times \left\langle \sum_{q\in\mathbb{Z}} v_{p,q} e^{-iq\alpha} \frac{w_{q,o}}{\|w_{q,o}\|}, \frac{w_{n,o}}{\|w_{n,o}\|} \right\rangle \frac{w_{m,o}(r\omega)}{\|w_{m,o}\|} \\
&= \sum_{m\in\mathbb{Z}} \sum_{n\in\mathbb{Z}} e^{i(n-m)\alpha} S(m,n,\beta) v_{p,n} e^{-in\alpha} \frac{w_{m,o}(r\omega)}{\|w_{m,o}\|} \\
&= \sum_{m\in\mathbb{Z}} \underbrace{\left( \sum_{n\in\mathbb{Z}} S(m,n,\beta) v_{p,n} \right)}_{\lambda_p v_{p,m}} e^{-im\alpha} \frac{w_{m,o}(r\omega)}{\|w_{w,o}\|} \\
&= \lambda_p \sum_{m\in\mathbb{Z}} v_{p,m} e^{-im(\alpha)} \frac{w_{m,o}(r\omega)}{\|w_{m,o}\|} \\
&= \lambda_p v_p(r\omega).
\end{aligned} \tag{51}$$

The normalization follows from

$$\begin{aligned}

1 &\overset{!}{=} \|v_p\|^2 \\

&= \sum_{m\in\mathbb{Z}} \sum_{n\in\mathbb{Z}} \left\langle v_{p,m} e^{-im\alpha} \frac{w_{m,o}}{\|w_{m,o}\|}, v_{p,n} e^{-in\alpha} \frac{w_{n,o}}{\|w_{m,o}\|} \right\rangle \\

&= \sum_{m\in\mathbb{Z}} |v_{p,m}|^2 = \|(v_{p,m})_{m\in\mathbb{Z}}\|^2_{l^2}.

\end{aligned} \tag{52}$$

To show that all eigenvectors are included, let $u$ be another eigenvector from $A^*A$ to the eigenvalue $\lambda$ and $u \neq v_p$ for all $p \in \mathbb{Z}$. Assume $u = \sum_{m\in\mathbb{Z}} \sum_{n\in\mathbb{N}} u_{m,n}(w_{m,n}/\|w_{m,n}\|)$ with respect to the basis $\{w_{m,n}\}_{m,n}$, then

$$\begin{aligned}

(A^*Au)(r\omega) &= \sum_{m\in\mathbb{Z}} \sum_{n\in\mathbb{Z}} e^{i(n-m)\alpha} S(m,n,\beta) \\

&\quad \times \left\langle \sum_{p\in\mathbb{Z}} \sum_{q\in\mathbb{N}} u_{p,q} \frac{w_{p,q}}{\|w_{p,q}\|}, \frac{w_{n,o}}{\|w_{n,o}\|} \right\rangle \frac{w_{m,o}(r\omega)}{\|w_{m,o}\|} \\

&= \sum_{m\in\mathbb{Z}} \sum_{n\in\mathbb{Z}} e^{i(n-m)\alpha} u_{n,o} S(m,n,\beta) \frac{w_{m,o}(r\omega)}{\|w_{m,o}\|}.

\end{aligned} \tag{53}$$

On the other hand,

$$A^*A\lambda u = \lambda \sum_{m\in\mathbb{Z}} \sum_{n\in\mathbb{Z}} u_{m,n} \frac{w_{m,n}}{\|w_{m,n}\|}. \tag{54}$$

By comparing the coefficients one can conclude that $u_{m,n} = 0$ for all $m \in \mathbb{Z}$ and $n \geq 1$. For the case $n = 0$, one can deduce

$$\begin{aligned}

\lambda u_{m,o} &= \sum_{n\in\mathbb{Z}} e^{i(n-m)\alpha} u_{n,o} S(m,n,\beta) \Longleftrightarrow \lambda e^{im\alpha} u_{m,o} \\

&= \sum_{n\in\mathbb{Z}} e^{in\alpha} u_{n,o} S(m,n,\beta).

\end{aligned} \tag{55}$$

It follows that $(e^{im\alpha}u_{m,o})_{m\in\mathbb{Z}}$ is an eigenvector of $(S(m,n,\beta))_{m,n}$. Therefore, one can choose $\tilde{p} \in \mathbb{Z}$, such that $(e^{im\alpha}u_{m,o})_{m\in\mathbb{Z}} = (v_{\tilde{p},m})_{m\in\mathbb{Z}}$ holds. It follows that

$$u = \sum_{m\in\mathbb{Z}} u_{m,o} \frac{w_{m,o}}{\|w_{m,o}\|} = \sum_{m\in\mathbb{Z}} e^{-im(\alpha)} v_{\tilde{p},m} \frac{w_{m,o}}{\|w_{m,o}\|} = v_{\tilde{p}}, \tag{56}$$

which is contradiction to $u \neq v_p$ for all $p \in \mathbb{Z}$.

Finally, the equation $Av_p = \sigma_p u_p$ has to hold for every $p \in \mathbb{Z}$. By using polar coordinates and the series representation of the Hankel function (see formula 10 in Appendix 7), one can conclude

$$\begin{aligned}

Av_p(R\omega_x) &= \frac{ik^2}{4} \sum_{m\in\mathbb{Z}} H_m(kR) Y_m(\omega_x) \\

&\quad \times \int_0^R r_y J_m(kr_y) \int_{S^1} \overline{Y_m(\omega_y)} v_p(r_y\omega_y) d\omega_y \, dr_y \\

&= \frac{ik^2}{4} \sum_{m\in\mathbb{Z}} H_m(kR) Y_m(\omega_x) \langle v_p, w_{m,o} \rangle_{L_2(\Omega)} \\

&= \frac{ik^2}{4} \sum_{m\in\mathbb{Z}} H_m(kR) Y_m(\omega_x) \|w_{m,o}\| v_{p,m} e^{-im\alpha}.

\end{aligned} \tag{57}$$

□

There are similarities to incomplete data problems in X-ray computerized tomography (CT), where the Sinc-matrix also played an important role for the SVD (see [13]). In CT it is well known that there exist a unique solution of the limited angle problem. Immediately the question arises, if there is also a unique solution in the inverse limited angle scattering problem at least for the radiating part of the source. Theorems 9 and 10 give a positive answer.

**Theorem 9.** *Let the parameters* $\alpha$ *and* $\beta$ *be fixed, let* $\{v_m^{\alpha,\beta}\}_{m\in\mathbb{Z}}$ *be the singular functions of Theorem 8 and* $\{w_{m,n}\}_{m\in\mathbb{Z},\, n\in\mathbb{N}}$ *the functions of the* $L_2$*-basis of Theorem 1. Then,*

(i) $\{v_m^{\alpha,\beta} \mid m \in \mathbb{Z}\}$ *is a complete orthogonal set of radiating sources,*

(ii) $\{w_{m,n} \mid m \in \mathbb{Z},\ 1 \le n \in \mathbb{N}\}$ *is a complete orthogonal set of nonradiating sources.*

*Proof.* Let

$$V_1 := \overline{\operatorname{span}\{v_m^{\alpha,\beta} \mid m \in \mathbb{Z}\}}, \qquad V_2 := \overline{\operatorname{span}\{w_{m,o} \mid m \in \mathbb{Z}\}}. \tag{58}$$

It is obvious that $V_1 \subseteq V_2$. Assume $x \in V_1^{C} \cap V_2$. Then, $x$ can be expanded in a series of functions from $V_2$, that is,

$$x = \sum_{m\in\mathbb{Z}} x_m \frac{w_{m,o}}{||w_{m,o}||}. \tag{59}$$

Let $P$ be the orthogonal projection onto $V_1$. We have the identity

$$\begin{aligned} 0 = Px &= \sum_{p\in\mathbb{Z}} \left\langle x, v_p^{\alpha,\beta}\right\rangle v_p^{\alpha,\beta} \\ &= \sum_{p\in\mathbb{Z}} \left\langle \sum_{q\in\mathbb{Z}} x_q \frac{w_{q,o}}{||w_{q,o}||}, \sum_{m\in\mathbb{Z}} v_{p,m}^{\beta} e^{-im\alpha} \frac{w_{m,o}}{||w_{m,o}||} \right\rangle v_p^{\alpha,\beta} \\ &= \sum_{p\in\mathbb{Z}} \left( \sum_{m\in\mathbb{Z}} x_m \overline{v_{p,m}^{\beta}} e^{im\alpha} \right) v_p^{\alpha,\beta}. \end{aligned} \tag{60}$$

Because the functions $v_p^{\alpha,\beta}$ are linear independent, this implies for all $p \in \mathbb{Z}$,

$$\begin{aligned} 0 &= \sum_{m\in\mathbb{Z}} x_m \overline{v_{p,m}^{\beta}} e^{im\alpha} \\ &= \left\langle (x_m e^{im\alpha})_{m\in\mathbb{Z}}, (v_{p,m}^{\beta})_{m\in\mathbb{Z}} \right\rangle. \end{aligned} \tag{61}$$

The vectors $(v_{p,m}^{\beta})_{m\in\mathbb{Z}} \in l_2$ build an orthonormal basis and the conclusion is

$$x_m e^{im\alpha} = 0 \Longleftrightarrow x_m = 0. \tag{62}$$

Hence $V_1 = V_2$ and Theorem 3 completes the proof. □

A direct consequence of the theorem above is that the data $g^{\alpha,\beta} = u_s^{\alpha}|_{\Gamma_{\alpha,\beta}}$ can be extended to the entire boundary $\partial\Omega$. Note that the minimum energy solution $\Phi_{\min}$ of the problem $A^{\alpha,\beta}\Phi = g^{\alpha,\beta}$ can be expressed with the help of the SVD by

$$\Phi_{\min} = \sum_{\sigma_m>0} \sigma_m^{-1} \left\langle g^{\alpha,\beta}, u_m^{\alpha,\beta}\right\rangle v_m^{\alpha,\beta}. \tag{63}$$

**Theorem 10.** *Let* $\alpha$, $\beta$ *be fixed and* $g^{\alpha,\beta} = u_s^{\alpha}|_{\Gamma_{\alpha,\beta}}$*. Then*

$$u_s^{\alpha}\big|_{\partial\Omega} = B\big|_{\partial\Omega}\Phi_{\min}, \tag{64}$$

*where* $\Phi_{\min}$ *is the minimum energy solution of* $A^{\alpha,\beta}\Phi = g^{\alpha,\beta}$*.*

*Proof.* The minimum energy solution is the projection of the source $\Phi^{\alpha}$ onto $N^{\perp}(A^{\alpha,\beta})$. But from Theorems 3 and 9 it follows that $N(A^{\alpha,\beta})^{\perp}$ is independent on $\beta$. Furthermore, we have $N(A^{\alpha,\beta})^{\perp} = N(B|_{\partial\Omega})^{\perp}$ which proves the statement. □

*Remark 1.* To extend the data on the entire boundary the minimum energy solution has to be computed. This problem is very ill-posed for small $\beta$, because numerical experiments have shown that $\lim_{\beta\to 0}\sigma_m^{\beta} = 0$ for all $m \in \mathbb{Z}$.

### 5. THE INVERSE PROBLEM

Many algorithms (see, e.g., [2, 14]) use the definition of the sources to reconstruct the unknown scatterer $f$. The problem can be divided into two separated steps. In a first step, the linear integral equation (11) has to be solved. This leads to the radiating parts of the sources $\Phi$. In a second nonlinear step, the nonradiating parts are to be computed.

### *5.1. The radiating parts of a source*

Because the minimum energy solution of the problem (11) is the unique solution in $N(A)^{\perp}$, this solution contains the radiating parts of the source $\Phi$. If the source is split into radiating and nonradiating parts $\Phi = \Phi_R + \Phi_{NR}$, the radiating part can be computed using the singular value decomposition $(v_m, u_m, \sigma_m)$ of the operator $A$. More precisely,

$$\Phi_R(r\omega) = \sum_m \sigma_m^{-1}\langle g, u_m\rangle v_m(r\omega). \tag{65}$$

The operator $A$ is a compact operator. Thus the problem is ill-posed in the following sense (see also [15]). Small errors in the data $g$ lead to large errors in the reconstruction $\Phi_R$. Hence a regularization has to be performed. A summary of regularization methods is given in [12]. All the methods mentioned in this book are closely related to a general framework, the approximate inverse (see [16–18]), where the basic idea is to approximate the solution $\Phi_R$ by smoothing

$$\begin{aligned} \Phi_R(x) \approx \Phi_{R,\gamma}(x) &:= \langle e_\gamma(x,\cdot), \Phi_R\rangle \\ &= \langle A^*\psi_\gamma(x,\cdot), \Phi_R\rangle = \langle \psi_\gamma(x,\cdot), g\rangle, \end{aligned} \tag{66}$$

here the mollifier $e_\gamma(x,\cdot)$ is an approximation of the delta-distribution (e.g., $e_\gamma(x,y) = (1/2\pi\gamma)e^{-\|x-y\|^2/2\gamma^2}$ the Gaussian) and the reconstruction kernel $\psi_\gamma(x,\cdot)$ is the solution of $A^*\psi_\gamma(x,\cdot) = e_\gamma(x,\cdot)$. The constant $\gamma$ denotes the regularization parameter, which depends on the noise level of the given data $g$. The advantages of this method are as follows.

(i) The reconstruction kernel $\psi_\gamma$ can be computed before the measurement process.

(ii) The kernel does not depend on the data, that are afflicted by errors.

(iii) If the same problem is to be solved many times (like the case for every given data $g^\alpha$), then the kernel $\psi_\gamma$ has to be computed only once. This fact yields a good performance.

Again we apply the SVD to get the kernel $\psi_\gamma^{\alpha,\beta}$,

$$\psi_\gamma^{\alpha,\beta}(x,\cdot) = \sum_{m=0}^{\infty} \sigma_m^{-1} \left\langle e_\gamma(x,\cdot), v_m^{\alpha,\beta} \right\rangle u_m^{\alpha,\beta}(\cdot). \tag{67}$$

For the numerical implementation one has to cut off the series. For $\gamma = 0$ which corresponds to the Delta—distribution and restricting the summation from $m = 0$ and up to $m = M$ that approach provides the truncated SVD.

A problem of the approximate inverse is the large memory consumption, because for every reconstruction point $x \in \Omega$ and for every incident wave direction $\alpha$ the kernel $\psi_\gamma^{\alpha,\beta}(x,\cdot)$ has to be computed. To overcome this problem, we use some invariance properties of the kernel (see also [18]).

*Remark 2.* If $\beta < \pi$, that is, $\Gamma$ is a real subset of $\partial\Omega$, no invariance has been found. In fact it is possible to extent the data on the entire boundary by now with the help of Theorem 10, but this also needs a regularization because of the ill-posedness of the problem. For this problem all reconstruction kernels have to be computed for every $\alpha$ and every reconstruction point $x$. At least the integrals $\langle e_\gamma(x,\cdot), w_{m,o}\rangle$ which appear in the inversion formula for the minimum energy solution of the reconstruction kernel

$$\psi_\gamma^{\alpha,\beta} = \sum_{p\in\mathbb{Z}} \frac{1}{\sigma_p^\beta} \sum_{m\in\mathbb{Z}} \frac{v_{p,m}^\beta e^{-im\alpha}}{\|w_{m,o}\|} \langle e_\gamma(x,\cdot), w_{m,o}\rangle u_p^{\alpha,\beta} \tag{68}$$

should be precomputed. This procedure still provides an acceptable performance of reconstructing the radiating parts of the source. In the following only the case $\beta = \pi$ is considered.

**Theorem 11.** *Let $\beta = \pi$ and $e_\gamma$ be a radial symmetric mollifier, that is, $e_\gamma(x, y) = e_\gamma(\|x - y\|)$. Then,*

(i) $\psi_\gamma^{\alpha,\pi}(re^{i\varphi_x}, Re^{i\varphi_y})$ *is independent of $\alpha$,*

(ii) $\psi_\gamma^{\pi}(re^{i\varphi_x}, \mathrm{R}e^{i\varphi_y}) = \psi_\gamma(r, \mathrm{R}e^{i(\varphi_x+\varphi_y)})$.

*Hence the kernel at any point $x = re^{i\varphi_x} \in \Omega$ is given by a rotation of the kernel at the point $r$ on the positive real axis.*

*Proof.* Let $\psi_\gamma^{\alpha,\pi}(r_xe^{i\varphi_x},\cdot)$ be the minimum energy solution of $A^*\psi_\gamma(r_xe^{i\varphi_x},\cdot) = e_\gamma(r_xe^{i\varphi_x},\cdot)$. Hence,

$$\psi_\gamma(r_xe^{i\varphi_x},\cdot) = \sum_m \sigma_m^{-1} \langle e_\gamma(r_xe^{i\varphi_x},\cdot), v_m\rangle u_m(\cdot). \tag{69}$$

Table 1: Reconstruction error of the radiating source.

| Data error $\|g_\epsilon\|/\|g\|$ in % | Reconstruction error $\|\Phi_R - \Phi_{\gamma,R}\|/\|\Phi_R\|$ |
|---|---|
| 0 | 0.0013 |
| 1 | 0.0044 |
| 2 | 0.0087 |
| 3 | 0.0131 |
| 4 | 0.0174 |
| 5 | 0.0218 |
| 6 | 0.0261 |
| 7 | 0.0305 |
| 8 | 0.0348 |
| 9 | 0.0392 |
| 10 | 0.0435 |

The inner products in (69) are given up to constants (see [7]) by

$$\begin{aligned}

&\langle e_\gamma(r_xe^{i\varphi_x},\cdot), w_{m,o}\rangle \\

&= \int_o^R r_yJ_m(kr_y) \int_o^{2\pi} \overline{Y_m(e^{i\varphi_y})} e_\gamma(\|r_xe^{i\varphi_x} - r_ye^{i\varphi_y}\|)\, d\varphi_y\, dr_y \\

&\overset{\varphi_y=\varphi_z-\varphi_x}{=} \int_o^R r_yJ_m(kr_y) \int_{\varphi_x}^{\varphi_x+2\pi} \overline{Y_m(e^{i(\varphi_z-\varphi_x)})} e_\gamma \\

&\qquad \times (\|r_xe^{i\varphi_x} - r_ye^{i(\varphi_z-\varphi_x)}\|)\, d\varphi_z\, dr_y \\

&= Y_m(e^{i\varphi_x}) \int_o^R r_yJ_m(kr_y) \int_{\varphi_x}^{\varphi_x+2\pi} \overline{Y_m(e^{i\varphi_z})} e_\gamma \\

&\qquad \times (\|e^{i\varphi_x}[r_x - r_ye^{i\varphi_z}]\|)\, d\varphi_z\, dr_y.

\end{aligned} \tag{70}$$

Since the function $\overline{Y_m(e^{i\varphi_z})}e_\gamma(\|\omega(\varphi_x)[r_x - r_ye^{i\varphi_z}]\|)$ is $2\pi$-periodic and $\|e^{i\varphi_x}\| = 1$ the inner products read as

$$\langle e_\gamma(r_xe^{i\varphi_x},\cdot), w_{m,o}\rangle = Y_m(e^{i\varphi_x}) \langle e_\gamma(r_x,\cdot), w_{m,o}\rangle. \tag{71}$$

□

With the approximate inverse it is possible to compute a smoothed solution of the radiating part of the source

$$\Phi_{R,\gamma}(re^{i\varphi}) = \langle g, D^{-\varphi}\psi(r,\cdot)\rangle, \tag{72}$$

where $D^\varphi$ denotes the rotation operator with respect to the angle $\varphi$. This method was tested in a numerical test for a homogeneous scatterer $f$, which is given by a disc with radius $\rho < R$. In this case the source and the field can be computed analytically, (see [19]). Table 1 shows the errors between the exact radiating source and the source reconstructed by the approximate inverse. Uniform noise $g_\epsilon$ where added to the exact data $g$. Different noise levels where added to the exact data. The regularization parameter $\gamma$ was adjusted to the data error.

For this example $\alpha$ is fixed and $\beta = \pi$. The radius is $R = 1.5$ and the wavenumber $k = 2\pi/0.9$. The object $f$ is a homogeneous disc with radius 0.5 and value 0.1.

### 5.2. The nonradiating parts of the sources $\Phi^\alpha$

Suppose that the data $g^{\alpha,\beta}$ are given for pairwise different incident waves $u_i^\alpha$. Then the radiating parts $\Phi_R^\alpha$ of the sources $\Phi^\alpha$ can be computed, as it was shown in Section 5.1. The nonradiating parts and the corresponding fields $u_{NR}^\alpha$ can be represented by the sets given in Theorems 3 and 5, leading to

$$\Phi_{NR}^\alpha(r\omega) = \sum_m \sum_{n\geq 1} a_{m,n}^\alpha w_{m,n}(r\omega),$$
$$u_{NR}^\alpha(r\omega) = \sum_m \sum_{n\geq 1} a_{m,n}^\alpha u_{m,n}(r\omega), \tag{73}$$

where the coefficients $a_{m,n}^\alpha$ are to be determined. To solve the problem numerically, the solution has to be calculated on a finite subspace. Two basic ideas which rely on [2] can be applied.

(1) Divide the problem into two parts. In the first step, the cost function

$$\sum_\alpha ||f(u_i^\alpha + B\Phi^\alpha) - \Phi^\alpha|| \tag{74}$$

has to be minimized by varying the scatterer $f$. In a second step, the cost functions

$$||f(u_i^\alpha + B\Phi_R^\alpha + u_{NR}^\alpha) - (\Phi_R^\alpha + \Phi_{NR}^\alpha)|| \tag{75}$$

for every given direction $\alpha \in S^1$ have to be minimized by variation of the coefficients $a_{m,n}^\alpha$ of the nonradiating parts $\Phi_{NR}^\alpha$ of the sources $\Phi^\alpha$. Return to the first step until changes in the profile are less than some specified tolerance. The two steps include a linear problem.

(2) Another idea is to solve the problem in a simultaneous way. As a consequence of (14) we have that $\Phi_{NR}^\alpha$ and $\Phi_{NR}^\beta$ are related by

$$\frac{(\Phi_R^\alpha + \Phi_{NR}^\alpha)}{(B\Phi_R^\alpha + B\Phi_{NR}^\alpha + u_i^\alpha)} - \frac{(\Phi_R^\beta + \Phi_{NR}^\beta)}{(B\Phi_R^\beta + B\Phi_{NR}^\beta + u_i^\beta)} = 0. \tag{76}$$

If the data $(g^{\alpha_k})_{k=1}^P$ are given for pairwise different incident waves $u_i^{\alpha_k}$, then the functional

$$||F(\Phi_{m,n}^{\alpha_k})_{m\in\mathbb{Z},\, n\geq 1}^{k=1,\ldots,P-1}||_{L_2^{P-1}(\Omega)}^2 \tag{77}$$

with

$$\begin{aligned}&\left(F(a_{m,n}^{\alpha_k})_{m\in\mathbb{Z},\, n\geq 1}^{k=1,\ldots,P-1}\right)_j \\ &= \frac{\Phi_R^{\alpha_j} + \sum_{m\in\mathbb{Z}}\sum_{n\geq 1} a_{m,n}^{\alpha_j} w_{m,n}}{B\Phi_R^{\alpha_j} + u_i^{\alpha_j} + \sum_{m\in\mathbb{Z}}\sum_{n\geq 1} a_{m,n}^{\alpha_j} u_{m,n}} \\ &\quad - \frac{\Phi_R^{\alpha_{j+1}} + \sum_{m\in\mathbb{Z}}\sum_{n\geq 1} a_{m,n}^{\alpha_{j+1}} w_{m,n}}{B\Phi_R^{\alpha_{j+1}} + u_i^{\alpha_{j+1}} + \sum_{m\in\mathbb{Z}}\sum_{n\geq 1} a_{m,n}^{\alpha_{j+1}} u_{m,n}}\end{aligned} \tag{78}$$

has to be minimized, for example, with the Gauss-Newton algorithm.

*Remark 3.* The iteration can also be performed for pairwise different wavenumbers $k$, because the scatterer $f$ does not depend on $k$ (see (14)).

### 6. NUMERICAL RESULTS

Figure 2 shows in the first row a complex valued scatterer. The real part is given on the left. The middle row shows the reconstruction of the scatterer. The data are given accurately. An error of 5% is added on the data for the reconstruction in the last row. The parameters of the problem and the measuring instrument are selected as follows: radius $R = 1$, wavenumber $k = 25, 128$ measuring directions equidistantly distributed between 0 and $2\pi$, and exactly as many measuring points per measuring direction. The scatterer $f$ consists of 5 single figures.

(i) Background circle: center $(0, 0)$, radius 0.7, value $0.05 + 0.05i$.
(ii) Lower circle: center $(0.2, -0.3)$, radius 0.125, value $0.1 + 0.075i$.
(iii) Middle circle: center $(0.2, 0)$, radius 0.1, value $0.2 + 0.175i$.
(iv) Upper circle: center $(0.2, 0.3)$, radius 0.075, value $0.3 + 0.275i$.
(v) Rectangle: right upper corner $(-0.1, 0.4)$, width 0.2, height 0.8, value $0.1 + 0.1i$.

The radiating parts of the source can be computed by solving linear systems of equations. The approximate inverse showed its ability to regularize this ill-posed problems in a fast way. The main advantages are listed in Section 5.1. The high memory consumption for the general case $\Gamma \subset \partial\Omega$ is still a problem. But because the time for solving the linear problems is negligible compared to the time for solving the nonlinear problem, Remark 2 shows an acceptable way out of this problem.

The nonradiating parts of the source were determined with the methods proposed in Section 5.2 and compared with one another. The quality of the reconstructions do not differ from one to the other. The linear algorithm needs more iterations than the simultaneous algorithm but less time per iteration. The speed strongly depends on the given parameters and the measuring setup. In fact, various approaches have been formulated, which try to solve the nonlinear inverse scattering problem with more or less approximations.

(i) Extended Born approximation, distorted wave Born approximation (DWBA) or Rytov approximation (see, e.g., [22–24]).
(ii) eikonal approximation method (see, e.g., [25]).
(iii) modified gradient method (MFG) (see, e.g., [26, 27]).
(iv) source-type integral equation method (STIE) (see, e.g., [2]).
(v) approximate inverse method (AI) (see, e.g., [7]).
(vi) propagation-backpropagation method (see, e.g., [28, 29]).

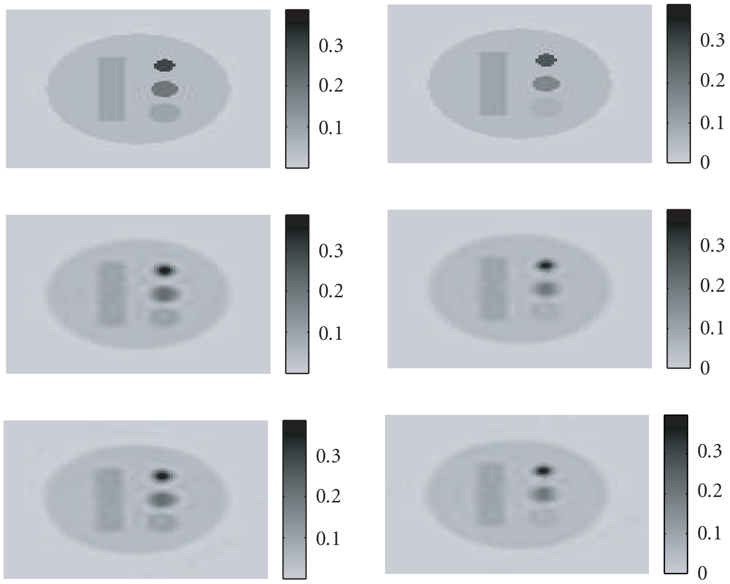

Figure 2: Reconstruction of a complex-valued scatterer.

(vii) sampling and probe method (see, e.g., [4, 30–33]).
(viii) contrast source inversion (CSI) (see, e.g., [14, 34]).
(ix) synthetic aperture focusing technique (SAFT) (see, e.g., [35]).

It is not the intention of the current paper to compare the performance of all reconstruction methods. It gives a better comprehension of the source-field system. Especially the solver for the linear problem has been improved by the approximate inverse.

### 7. CONCLUSION

In the first section of this paper, a special orthogonal basis of the space $L_2(\Omega)$ for $\Omega$ a unit ball has been derived. Using this basis it is possible to separate the source in its radiating and nonradiating parts, respectively. The radiating parts of a source can be obtained by solving an ill-posed linear problem. In our paper, they are computed by means of the approximate inverse. A numerical example for a known scatterer and different noise levels shows the applicability of the method. The nonradiating parts can be calculated by nonlinear optimization. The theoretical results are verified in Section 6 by numerical experiments, indicating that the here presented method is applicable for the problem under consideration.

### APPENDICES

### APPENDIX A

Lommel's theorem provides a useful tool to construct an orthogonal system consisting of Bessel functions.

**Theorem A.1** (*Lommel*). *Let $0 < x < y$ and $\lambda, \mu \in \mathbb{C} \setminus \{0\}$. Let $\nu \in \mathbb{C}$ and*

$$h_\nu(z) = aJ_\nu(\lambda z) + bN_\nu(\lambda z),$$
$$g_\nu(z) = cJ_\nu(\mu z) + dN_\nu(\mu z), \quad \text{where } a, b, c, d \in \mathbb{C}. \tag{A.1}$$

(i) *If $\mu^2 \neq \lambda^2$, then*

$$\begin{aligned}\int_x^y z h_\nu(z) g_\nu(z)\,dz &= \frac{z}{\lambda^2 - \mu^2}\left(h'_\nu(z)g_\nu(z) - h_\nu(z)g'_\nu(z)\right)\Big|_x^y \\ &= \frac{z}{\lambda^2 - \mu^2}\left(\lambda h_{\nu+1}(z)g_\nu(z) - \mu h_\nu(z)g_{\nu+1}(z)\right)\Big|_x^y.\end{aligned} \tag{A.2}$$

(ii) *If $\mu^2 = \lambda^2$, then*

$$\int_x^y z h_\nu(z) g_\nu(z)\,dz = \frac{1}{2\lambda^2}\left(z^2 h'_\nu(z)g'_\nu(z) + (\lambda^2 z^2 - \nu^2)h_\nu(z)g_\nu(z)\right)\Big|_x^y. \tag{A.3}$$

This is shown, for example, in [8]. Because the Bessel functions of the first kind are bounded near the origin the following special case of Lommel's theorem holds true.

**Theorem A.2.** *Let $a \in \mathbb{C}$, $m \in \mathbb{Z}$ and*

(i) *$\lambda, \mu \in \mathbb{C} \setminus \{0\}$ zeros of $J_m$. Then,*

$$\begin{aligned}&\int_0^1 rJ_m(\lambda r)J_m(\mu r)\,dr = 0, \quad \text{if } \lambda^2 \neq \mu^2, \\ &\int_0^1 rJ_m^2(\lambda r)\,dr = \frac{1}{2\lambda^2}\left[J'_m(\lambda)\right]^2 \quad \text{else.}\end{aligned} \tag{A.4}$$

Table 2

| | | | |
|---|---|---|---|
| | (1) Bessel functions of the first kind $J_\nu$ | | |
| 1 | The zeros of $J_\nu$ are real | $\nu > -1$ | [8] |
| 2 | The zeros of $aJ_\nu(z) + zbJ'_\nu(z)$ are real | $\nu > -1, 0 \neq b \in \mathbb{R}$ $a \in \mathbb{R}, \frac{a}{b} + \nu \geq 0$ | [20] |
| 3 | The zeros of $aJ_\nu(z) + zbJ'_\nu(z)$ are real beside of two pure imaginary zeros | $\nu > -1, 0 \neq b \in \mathbb{R}$ $a \in \mathbb{R}, \frac{a}{b} + \nu < 0$ | [20] |
| 4 | The positive zeros of $aJ_\nu(z) + zbJ'_\nu(z)$ and $cJ_\nu(z) + zdJ'_\nu(z)$ are interlaced | $\nu > -1, a, b, c, d \in \mathbb{R}$ $ad \neq bc$ | [8] |
| 5 | The positive zeros of $J_\nu$ are countable $\lambda_0 < \lambda_1 < \dots$, where $\lim_{n\to\infty} \lambda_n = \infty$ | $\nu \in \mathbb{R}$ | [21] |
| 6 | $J_\nu$ is bounded near the origin | $\mathrm{Re}(\nu) \geq 0$ | [21] |
| 7 | $J_m(0) = 0$ | $m \in \mathbb{Z} \setminus \{0\}$ | [21] |
| | (2) Bessel functions of the second kind $N_\nu$ | | |
| 8 | $N_\nu(z) = \frac{J_\nu(z)\cos(\nu\pi) - J_{-\nu}(z)}{\sin(\nu\pi)}$ | $\nu \notin \mathbb{Z}, z \in \mathbb{C}$ | [21] |
| | $= \lim_{\mu\to\nu} \frac{J_\mu(z)\cos(\mu\pi) - J_{-\mu}(z)}{\sin(\mu\pi)}$ | $\nu \in \mathbb{Z}, z \in \mathbb{C}$ | [21] |
| | (3) Hankel functions $H_\nu$ (Bessel function of the third kind) | | |
| 9 | $H_\nu(z) = J_\nu(z) + iN_\nu(z)$ | $\nu, z \in \mathbb{C}$ | [21] |
| 10 | $H_0(k\|x-y\|) = \sum_{m\in\mathbb{Z}} H_m(k\|x\|) Y_m\left(\frac{x}{\|x\|}\right) J_m(k\|y\|) \overline{Y_m\left(\frac{y}{\|y\|}\right)}$ | $\|x\| > \|y\|$ | [21] |
| 11 | $J_{\nu+1}(z)N_\nu(z) - J_\nu(z)N_{\nu+1}(z) = \frac{2}{\pi z}$ | $\nu \in \mathbb{Z}, z \in \mathbb{C}$ | [21] |

(ii) $\lambda, \mu \in \mathbb{C} \setminus \{0\}$ *zeros of* $aJ_m(r) + rJ'_m(r)$. *Then holds*

$$\int_0^1 rJ_m(\lambda r)J_m(\mu r)dr = 0, \quad \text{if } \lambda^2 \neq \mu^2,$$

$$\int_0^1 rJ_m^2(\lambda r)dr = \frac{1}{2\lambda^2}[a^2 + \lambda^2 - m^2]J_m^2(\lambda) \quad \text{else.} \tag{A.5}$$

### APPENDIX B

Some useful properties of Bessel functions are summarized in Table 2.

### REFERENCES

[1] P. M. Morse and H. Feshbach, *Methods of Theoretical Physics, Part I and II*, McGraw-Hill, New York, NY, USA, 1953.

[2] T. M. Habashy, M. L. Oristaglio, and A. T. de Hoop, "Simultaneous nonlinear reconstruction of two-dimensional permittivity and conductivity," *Radio Science*, vol. 29, no. 4, pp. 1101–1118, 1994.

[3] A. Devaney and G. Sherman, "Nonuniqueness in inverse source and scattering problems," *IEEE Transactions on Antennas and Propagation*, vol. 30, no. 5, pp. 1034–1037, 1982.

[4] D. Colton and R. Kress, *Inverse Acoustic and Electromagnetic Scattering Theory*, Springer, Heidelberg, Germany, 1992.

[5] F. Hettlich and A. Kirsch, "Schiffer's theorem in inverse scattering theory for periodic structures," *Inverse Problems*, vol. 13, no. 2, pp. 351–361, 1997.

[6] A. Gamliel, K. Kim, A. I. Nachman, and E. Wolf, "A new method for specifying nonradiating, monochromatic, scalar sources and their fields," *Journal of the Optical Society of America*, vol. 6, no. 9, pp. 1388–1393, 1989.

[7] H. Abdullah and A. K. Louis, "The approximate inverse for solving an inverse scattering problem for acoustic waves in an inhomogeneous medium," *Inverse Problems*, vol. 15, no. 5, pp. 1213–1229, 1999.

[8] G. N. Watson, *A Treatise on the Theory of Bessel Functions*, Cambridge Mathematical Library, Cambridge University Press, Cambridge, UK, 1958.

[9] H. Hochstadt, *The Functions of Mathematical Physics*, Dover Books on Physics and Chemistry, Dover, New York, NY, USA, 1986.

[10] E. Wallacher, "Äquivalente Quellen als Zugang zur Lösung eines inversen Streuproblems," M.S. thesis, Universität des Saarlandes, Saarbrücken, Germany, 2002.

[11] E. A. Marengo and R. W. Ziolkowski, "Nonradiating and minimum energy sources and their fields: generalized source inversion theory and applications," *IEEE Transactions on Antennas and Propagation*, vol. 48, no. 10, pp. 1553–1562, 2000.

[12] A. K. Louis, *Inverse und Schlecht Gestellte Probleme*, Teubner, Stuttgart, Germany, 1989.

[13] A. K. Louis, "Incomplete data problems in X-ray computerized tomography I. Singular value decomposition of the limited angle transform," *Numerische Mathematik*, vol. 48, no. 3, pp. 251–262, 1986.

[14] A. Abubakar, P. M. van den Berg, and T. M. Habashy, "Application of the multiplicative regularized contrast source inversion method on TM- and TE-polarized experimental Fresnel data," *Inverse Problems*, vol. 21, no. 6, pp. S5–S13, 2005.

[15] J. Hadamard, *Lectures on Cauchy's Problem in Linear Partial Differential Equations*, Yale University Press, New Haven, Conn, USA, 1923.

[16] A. K. Louis and P. Maaß, "A mollifier method for linear operator equations of the first kind," *Inverse Problems*, vol. 6, no. 3, pp. 427–440, 1990.

[17] A. K. Louis, "Approximate inverse for linear and some nonlinear problems," *Inverse Problems*, vol. 12, no. 2, pp. 175–190, 1996.

[18] A. K. Louis, "Constructing an approximate inverse for linear and some nonlinear problems in engineering," in *Proceedings of the 1996 2nd International Conference on Inverse Problems in Engineering: Theory and Practice*, pp. 367–374, American Society of Mechanical Engineers, Le Croisic, France, June 1998.

[19] F. Wübbeling, *Das direkte und das inverse Streuproblem bei fester Frequenz*, Ph.D. thesis, Universität Münster, Münster, Germany, 1995, 4/95-N.

[20] A. C. Dixon, "On a property of Bessel's functions," *Messenger*, 1903.

[21] M. Abramowitz and I. A. Stegun, *Handbook of Mathematical Functions with Formulas, Graphs, and Mathematical Table*, Dover, New York, NY, USA, 1965.

[22] T. M. Habashy, R. W. Groom, and B. R. Spies, "Beyond the Born and Rytov approximations: a nonlinear approach to electromagnetic scattering," *Journal of Geophysical Research*, vol. 98, no. B2, pp. 1759–1775, 1993.

[23] Y. Fei and A. J. Devaney, "Inverse scattering for real-valued scattering potentials," *Inverse Problems*, vol. 21, no. 4, pp. L7–L12, 2005.

[24] T. J. Cui, Y. Qin, G.-L. Wang, and W. C. Chew, "Low-frequency detection of two-dimensional buried objects using high-order extended Born approximations," *Inverse Problems*, vol. 20, no. 6, pp. S41–S62, 2004.

[25] A. K. Louis, "The eikonal approximation in ultrasound computer tomography," in *Proceedings of the IMA Conference on Signal Processing*, 1989.

[26] R. E. Kleinman and P. M. van den Berg, "A modified gradient method for two-dimensional problems in tomography," *Journal of Computational and Applied Mathematics*, vol. 42, no. 1, pp. 17–35, 1992.

[27] G. Pelekanos, R. E. Kleinman, and P. M. van den Berg, "Inverse scattering in elasticity - a modified gradient approach," *Wave Motion*, vol. 32, no. 1, pp. 57–65, 2000.

[28] F. Natterer and F. Wubbeling, "A propagation-backpropagation method for ultrasound tomography," *Inverse Problems*, vol. 11, no. 6, pp. 1225–1232, 1995.

[29] M. Vögeler, "Reconstruction of the three-dimensional refractive index in electromagnetic scattering by using a propagation-backpropagation method," *Inverse Problems*, vol. 19, no. 3, pp. 739–753, 2003.

[30] R. Potthast, "A survey on sampling and probe methods for inverse problems," *Inverse Problems*, vol. 22, no. 2, pp. R1–R47, 2006.

[31] B. Gebauer, M. Hanke, A. Kirsch, W. Muniz, and C. Schneider, "A sampling method for detecting buried objects using electromagnetic scattering," *Inverse Problems*, vol. 21, no. 6, pp. 2035–2050, 2005.

[32] A. Kirsch, "The factorization method for Maxwell's equations," *Inverse Problems*, vol. 20, no. 6, pp. S117–S134, 2004.

[33] M. Ikehata, "A new formulation of the probe method and related problems," *Inverse Problems*, vol. 21, no. 1, pp. 413–426, 2005.

[34] C. Yu, L.-P. Song, and Q. H. Liu, "Inversion of multi-frequency experimental data for imaging complex objects by a DTA-CSI method," *Inverse Problems*, vol. 21, no. 6, pp. S165–S178, 2005.

[35] R. Marklein, K. Mayer, R. Hannemann, et al., "Linear and nonlinear inversion algorithms applied in nondestructive evaluation," *Inverse Problems*, vol. 18, no. 6, pp. 1733–1759, 2002.

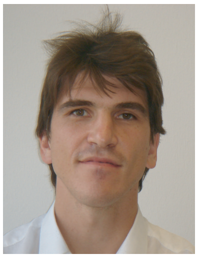

**E. Wallacher** received his Diploma in mathematics in 2002 from Saarland University in Germany. Since then, he is working as a Research Assistant at the Chair of Applied Mathematics held by Professor A. K. Louis. His research interests are in the regularization of ill-posed problems and in scattering theory, which will also be the topic of his Ph.D. thesis.

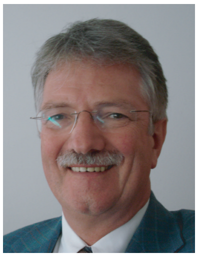

**A. K. Louis** received his Diploma in applied mathematics at Saarland University, in 1972, his Ph.D. in Mainz, in 1972, and the Habilitation in Münnster 1982. The academic year 1980–1981 he spent as an Assistant Professor at the State University of New York, in Buffalo. From 1983–1986, he was Associate Professor at the University in Kaiserslautern; from 1986–1990, he had a position as a Full Professor at the Technical University of Berlin. Since 1990, he has the Chair for Applied mathematics at Saarland University in Saarbrücken. He declined offers from the university in Munich and Louisiana State University at Baton Rouge. He was Vice-President of the Saarland University in 1997–1999. He is Chevalier dans l'Ordre de la Palme Académique. Since 1978, he is working on mathematical problems from computerized tomography. He published books on Inverse Problems and on Wavelets, he organized several international conferences on inverse problems and on medical imaging.

